# Functional imaging in combination with mutation status aids prediction of response to inhibiting B-cell receptor signaling in lymphoma

**DOI:** 10.18632/oncotarget.20551

**Published:** 2017-08-24

**Authors:** Laura Jacobs, Stefan Habringer, Jolanta Slawska, Katharina Huber, Elke Hauf, Zhoulei Li, Yosef Refaeli, Markus Schwaiger, Martina Rudelius, Axel Walch, Ulrich Keller

**Affiliations:** ^1^ Nuclear Medicine Department, Technische Universität München, Munich, Germany; ^2^ Internal Medicine III, Technische Universität München, Munich, Germany; ^3^ Analytical Pathology, Helmholtz Zentrum München, Neuherberg, Germany; ^4^ Department of Dermatology, University of Colorado, Denver, CO, USA; ^5^ Department of Pathology, Universitätsklinikum Düsseldorf, Düsseldorf, Germany; ^6^ German Cancer Consortium (DKTK) and German Cancer Research Center (DKFZ), Heidelberg, Germany

**Keywords:** lymphoma, B-cell receptor signaling, positron emission tomography, MALDI imaging mass spectrometry, functional imaging

## Abstract

Aberrant B-cell receptor (BCR) signaling is known to contribute to malignant transformation. Two small molecule inhibitors targeting BCR pathway signaling include ibrutinib, a Bruton’s tyrosine kinase (BTK) inhibitor, and idelalisib, a specific Phosphatidylinositol-4,5-bisphosphate 3-kinase delta (PI3Kδ) inhibitor, both of which have been approved for use in haematological malignancies. Despite the identification of various diffuse large B-cell lymphoma (DLBCL) subtypes, mutation status alone is not sufficient to predict patient response and therapeutic resistance can arise. Herein we apply early molecular imaging across alternative activated B-cell (ABC) and germinal center B-cell (GCB) DLBCL subtypes to investigate the effects of BCR pathway inhibition. Treatment with both inhibitors adversely affected cell growth and viability. These effects were partially predictable based upon mutation status. Accordingly, very early 2-deoxy-2-[18F]fluoro-D-glucose positron emission tomography (^18^F-FDG-PET) and 3’-deoxy-3’[18F]-fluorothymidine positron emission tomography (^18^F-FLT-PET) reported tumour regression and reductions in tumour metabolism and proliferation upon treatment. Furthermore, matrix-assisted laser desorption ionization imaging mass spectrometry (MALDI IMS) identified alterations in the proteome of a model of ABC DLBCL upon treatment with ibrutinib or idelalisib. In conclusion we demonstrate that very early molecular imaging adds predictive value in addition to mutational status of DLBCL that may be useful in directing patient therapy.

## INTRODUCTION

Diffuse large B-cell lymphomas (DLBCL) are a heterogeneous group of non-Hodgkin’s lymphomas (NHL) accounting for 30-40% of newly diagnosed lymphoma cases with clinical outcome strongly dependent upon subtype and risk factors at diagnosis [1]. Activated B-cell (ABC) DLBCL, known to be ‘addicted’ to NF-kappaB (NF-κB) signaling, may have a poorer therapeutic prognosis than germinal center B-cell (GCB) lymphomas expressing genes normally found in germinal center B-cells [2]. Unlike GCB DLBCL it is established that ABC DLBCL frequently rely upon B-cell receptor (BCR) signaling for sustained survival [3]. It is generally recognized that BCR signaling is antigen independent (tonic) or antigen dependent (active). In ABC DLBCL the BCR signaling mode has been more specifically described as ‘chronic active’, being qualitatively similar to the signaling of the antigen exposed B-cell [4]. The predominant pathway activations include CARD11 NF-κB-dependent activation in ∼10% of cases [5] and Myd88 mutations observed in ∼39% of cases [6], both contributing to constitutive NF-κB activity in ABC DLBCL.

A variety of drugs have been developed to address these different BCR signaling subtypes [7], however due to relapse following DLBCL therapy and the emergence of mutational resistance a number of small molecule inhibitors have been developed. As an example the Bruton’s tryosine kinase (BTK) inhibitor ibrutinib irreversibly binds BTK and elicits a potent effect, with ∼40% of DLBCL patients responsive to therapy [8]. Alternatively, idelalisib (also CAL-101 or GS-1101) targets the phosphoinositide 3-kinase (PI3K) pathway acting to disrupt key cellular processes including metabolism and cell growth [9]. Despite idelalisib being established in the treatment of chronic lymphocytic leukaemia (CLL) and other indolent B-cell NHL [10], its utility is still under investigation in the context of aggressive lymphoma [11].

It is known that variability in DLBCL treatment response can be reflected by mutation status across subtypes, characterized by mutations targeting genes for specific subunits of the BCR signaling pathway such CD79a/b [8, 12, 13]. Previously histopathology has been used to define DLBCL and next generation sequencing has been instrumental in identifying novel characteristic mutations, despite this tumors can exhibit further heterogeneity in protein expression and post translational modifications (PTMs) profiles [14] contributing to the complexity of such disease. Despite established imaging methods such as ^18^F-FDG-PET facilitating assessment of therapy response and prediction of disease-free survival [15, 16], emerging molecular imaging methods such as matrix-assisted laser desorption ionization imaging mass spectrometry (MALDI IMS) are now able to identify proteins, metabolites and also small molecule drugs *in situ* [17]. Application of an acidic matrix to a tissue section, followed by laser desorption to ionize proteins that are then detected using mass spectrometry, provides in-depth spatial information of the whole tissue surface. Examples of MALDI IMS application in tissue studies extends from breast cancer classification [18] to markers of metastatic melanoma recurrence [19] and drug detection [20, 21].

In light of the reported promise of the BCR inhibitors ibrutinib and possibly also idelalisib in treatment of molecularly selected DLBCL, we address herein the question of whether an early response to BCR-directed therapy can be observed using a combination of molecular imaging techniques and mutational status in DLBCL.

## RESULTS

### The mutation status of specific BCR pathway components predicts the *in vitro* response to BCR inhibition

**Figure 1:**
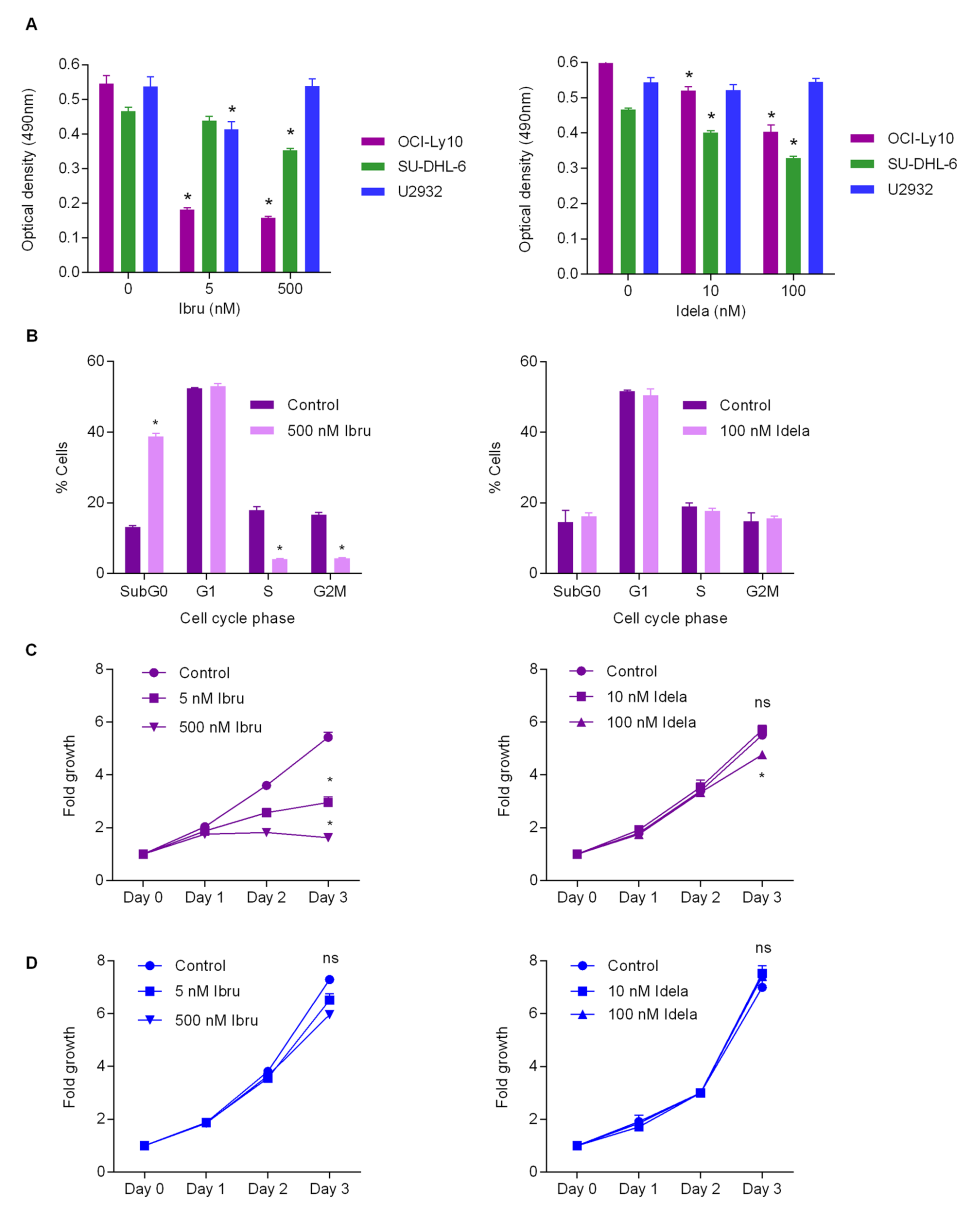
Mutational status predicts BCR inhibition response *in vitro* **(A)** Optical density for OCI-LY10, U-2932 and SU-DHL-6 cells following ibrutinib (*left*) and idelalisib (*right*) treatment as measured by MTT assay. Values reported as optical density adjusted for background, error bars represent standard deviation (SD) (n=4). Student’s t-test was used to compare treated values versus control cells. Asterisks indicate statistical significance (p < 0.05). **(B)** Cell cycle analysis of ibrutinib treated OCI-LY10 (*left*) and idelalisib treated OCI-LY10 (*right*). Values reported as percentage of cells analyzed, error bars represent SD (n=3). Asterisks indicate statistical significance (p < 0.05). **(C)** Growth curve analysis of OCI-LY10 cells in response to ibrutinib (*left*) or idelalisib (*right*). Error bars represent SD (n=4). Asterisks indicate statistical significance (p < 0.05). **(D)** Growth curve analysis of U2932 cells in response to ibrutinib (*left*) or idelalisib (*right*). Error bars represent SD (n=3). Asterisks indicate statistical significance (p < 0.05).

It is established that the mutational status of DLBCL can be predictive of therapy outcome when addressed using specific treatment regimens [8, 12, 13]. In order to establish whether mutational status of lymphoma cells *in vitro* is predictive of response to the BCR inhibitors ibrutinib and idelalisib, we tested their effect on the ABC DLBCL cell lines OCI-LY10, U-2932 and also on the GBC DLBCL line SU-DHL-6 that is not anticipated to be affected by the BTK inhibitor ibrutinib, but potentially by PI3K inhibition using idelalisib. As OCI-LY10 and U-2932 cells carry BCR pathway mutations – in CD79A ITAM/Myd88b and TAK1 respectively - it was hypothesized that differences in ability of the inhibitors to affect cell viability would be observed. Indeed, OCI-LY10 cells carrying the upstream mutation was responsive to both inhibitors demonstrating a significant drop in cell viability upon treatment with all concentrations of ibrutinib and idelalisib compared to controls (Figure 1A). SU-DHL-6 cells also responded significantly to both ibrutinib and idelalisib treatment at all higher doses. In the case of idelalisib treatment U-2932 cells basically did not respond to inhibition as compared to OCI-LY10 cells, whereas SU-DHL-6 demonstrated a similar reduction in viability upon idelalisib treatment (Figure 1A). Cell cycle analysis indicated that idelalisib treatment had no significant effect on cell cycle status of OCI-LY10 cells, however a significant increase of sub-G0 phase and a significant decrease in G1 phase to the highest dose of ibrutinib could be observed (Figure 1B) indicating a reduction in cell growth. Furthermore ibrutinib treatment induced a significant decrease in S and G2M phase activity in response to treatment (Figure 1B). Growth curve analysis of OCI-LY10 cells revealed a modest but significant response to idelalisib treatment at the highest dose, and significant decreases in growth in response to ibrutinib treatment (Figure 1C). Conversely U-2932 demonstrated no significant response to either ibrutinib or idelalisib upon treatment (Figure 1D). Together these data support that mutational status can in part be used to predict therapy response to BCR pathway inhibition *in vitro*.

### Early therapy response is observed following BCR inhibition via ^18^F-FDG-PET and ^18^F-FLT-PET imaging

**Figure 2:**
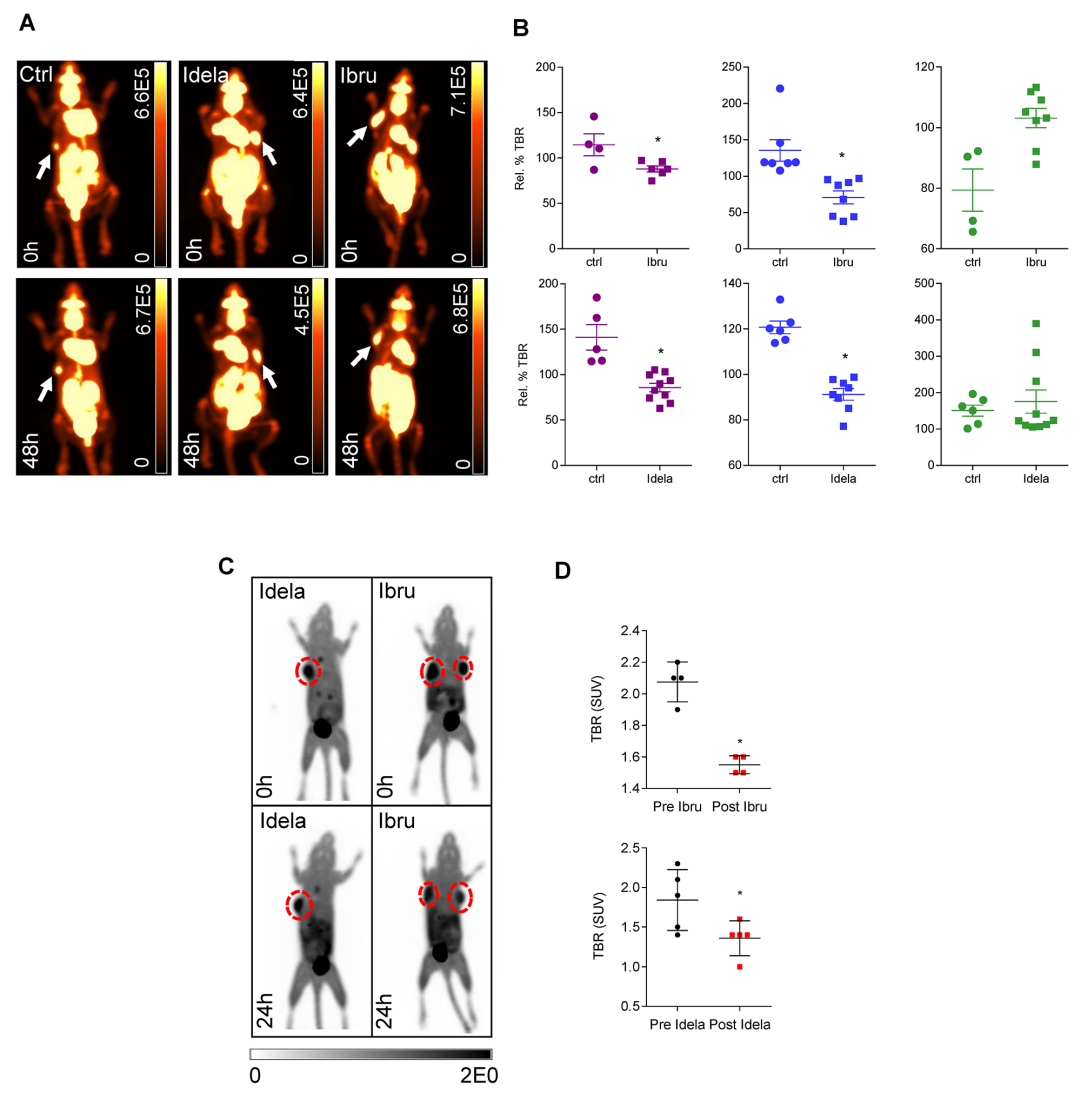
Therapeutic response to idelalisib or ibrutinib can be observed at an early time point *in vivo* **(A)** ^18^F-FDG-PETimages of OCI-LY10 xenografts in NOD SCID mice without treatment (*left*) idelalisib treatment (*middle)* or ibrutinib treatment (*right*). All images 48 hours post treatment. Scale bars: Bq/ml. **(B)** Response of OCI-LY10 (*left*) U-2932 (*middle*) and SU-DHL-6 (*right*) xenografts to ibrutinib (*upper panel*) or idelalisib treatment (*lower panel*) *in vivo* as measured by relative percentage tumour to background (Rel. % TBR) ratio at 48 hours post treatment. Student’s t-test, *p < 0.05. **(C)** ^18^F-FLT-PET imaging of OCI-LY10 xenografts demonstrated reduced ^18^F-FLT uptake following BCR inhibition. Mice were imaged, treated with ibrutinib or idelalisib for 48 hours and imaged post-treatment. Scale bars standard uptake values (SUV). **(D)** TBR derived from standard uptake values (SUV) for OCI-LY10 xenografts following ibrutinib (n = 4) and idelalisib treatment (n = 5) treatment. Student’s t-test, *p < 0.05.

**Figure 3:**
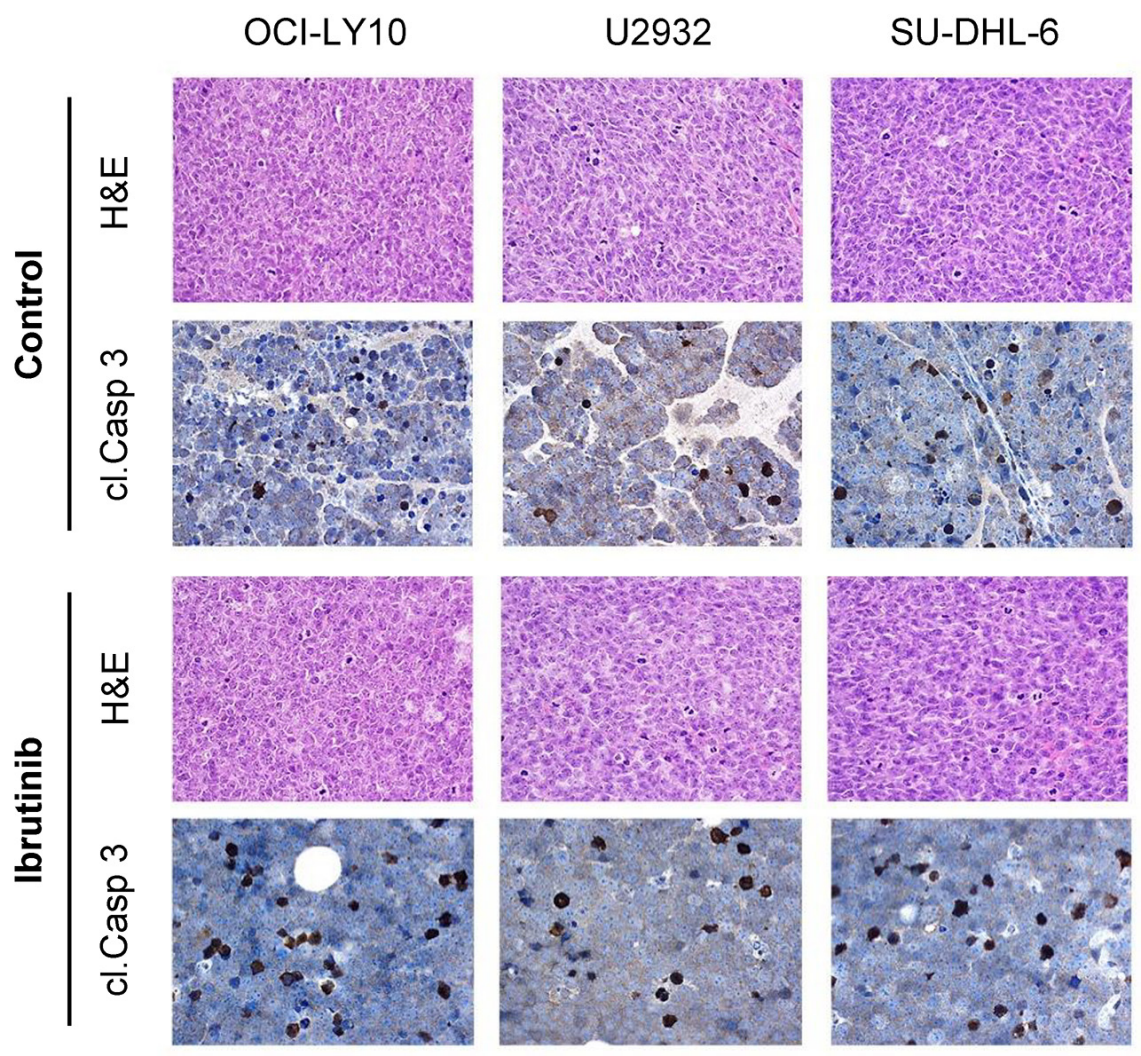
Tumour response to ibrutinib treatment reflects mutational status Representative H&E stains of tumour xenografts including cleaved caspase-3 (cl. Casp 3) are shown for each cell line (OCI-LY10, U2932, SU-DHL-6) in the untreated and ibrutinib treated state (magnification 200x).

Following the observation of the effect of mutation status upon *in vitro* treatment response to BCR pathway inhibitors, we next aimed to establish whether ^18^F-FDG-PET imaging of lymphoma xenografts could be used to predict therapy response to BCR inhibition *in vivo* (Figure 2A). Results demonstrated that tumour-background ratios (TBRs) of OCI-LY10 xenografts in mice were reduced following both ibrutinib and idelalisib treatment (Figure 2B, *left panel*), indicating a reduction in tumour growth and glucose metabolism. Unexpectedly, U-2932 xenografts also displayed a reduction in TBR following treatment with both inhibitors compared to controls (Figure 2B, *middle panel*), however SU-DHL-6 xenografts were affected by neither ibrutinib nor idelalisib (Figure 2B, *right panel*), reflecting their reported lack of dependence upon BCR pathway signaling. Furthermore the reduction in TBR of OCI-LY-10 xenografts upon ^18^F-FDG-PET imaging was supported by a reduction in ^18^F-FLT signal (Figure 2C) and calculated TBR values (Figure 2D) following treatment with both inhibitors, indicating a reduced proliferation also. Immunohistochemistry of ibrutinib treated xenografts confirmed that treatment response reflects mutational status (Figure 3) with a strong trend towards an increase in apoptosis in ibrutinib treated OCI-LY10 xenografts.

**Figure 4:**
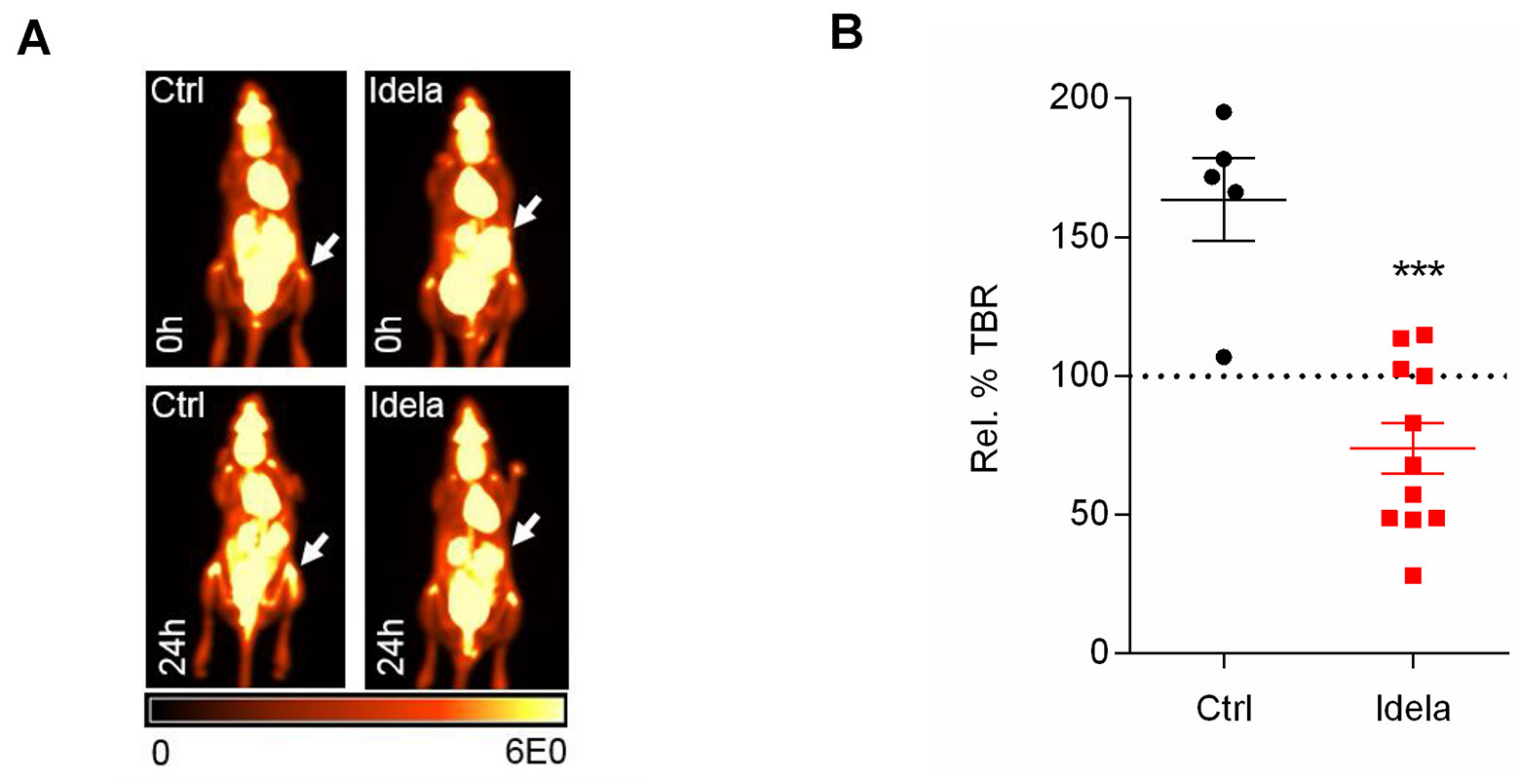
Tonic BCR signaling in lymphoma **(A)** Idelalisib treatment in E*μ-Myc*/BCR^HEL^/sHEL syngeneic transplantation model demonstrates early splenic treatment response *in vivo*. Representative ^18^F-FDG-PET images of mice pre and 48 hours post treatment. Values reported as percentage injected dose per gram body weight (%ID/g). **(B)** Percentage relative TBRs of E*μ-Myc*/BCR^HEL^/sHEL spleens following idelalisib treatment (n = 5-11).

To further explore early treatment response to idelalisib *in vivo*, the syngeneic transplantation model E*μ-Myc*/BCR^HEL^/sHEL representing tonic BCR signaling with PI3K activation was used [22]. Mice were imaged with ^18^F-FDG-PET before and after treatment with idelalisib (Figure 4A). As observed for the DLBCL xenografts an early treatment response was reported with a significant reduction in splenic TBR at 48 hours post therapy (Figure 4B), supporting that the PI3K inhibitor idelalisib can be an effective treatment in the treatment of tonic B-cell receptor signaling.

### MALDI IMS proteomic analysis of BCR inhibition response

Upon establishing that ^18^F-FDG-PET imaging can be used to monitor BCR therapy response at very early time points and noting the key role of BCR signaling in DLBCL survival, we postulated that a distinctive protein profile may manifest in tumours following treatment with different BCR pathway inhibitors. Using a MALDI IMS proteomics approach we profiled the tumours treated with ibrutinib and idelalisib *in vivo*. As the mutational status of the OCI-LY10 cell line lends itself to response to both inhibitors we performed proteomic analysis on xenografts derived from these cells.

**Figure 5:**
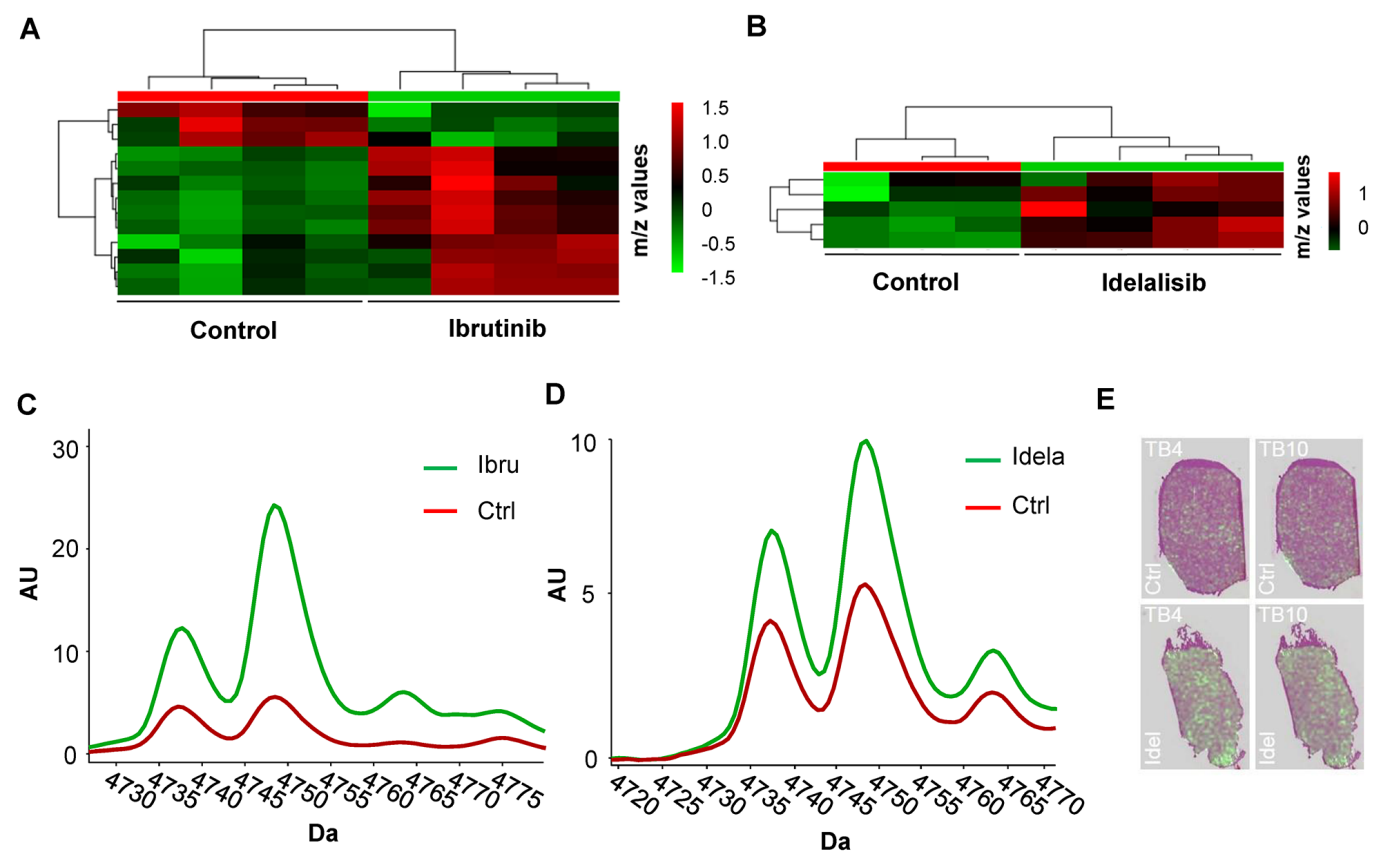
MALDI proteomic analysis of OCI-LY10 xenografts treated with ibrutinib and idelalisib **(A)** Proteomic heatmap of control versus ibrutinib treated OCI-LY10 tumours displays clustering (n= 4). **(B)** Proteomic heatmap of control versus idelalisib treated OCI-LY10 tumours displays clustering (n= 3-4). **(C)** Profiles of thymosin beta-4 and thymosin beta-10 levels in OCI-LY10 xenografts treated with ibrutinib postulated peak identities; m/z 4738 = Thymosin beta-10, m/z 4749 = Thymosin beta-4 (-2 AA’s, cleaved to seraspenide), n = 6. **(D)** Profiles of thymosin beta-4 and thymosin beta-10 levels in OCI-LY10 xenografts treated with idelalisib postulated peak identities; m/z 4738 = Thymosin beta-10, m/z 4749 = Thymosin beta-4 (-2 AA’s, cleaved to seraspenide), n = 6. **(E)** Representative overlays of distribution of thymosin beta-4 and thymosin beta-10 proteins on H & E sections in control and idelalisib treated OCI-LY10 xenografts.

Upon treatment with ibrutinib an overall deregulation of proteins could be observed between treated and untreated xenografts (Figure 5A). The same proteomic approach was used following idelalisib treatment and resulted in fewer deregulation events upon treatment (Figure 5B), however alterations in the expression profile of proteins detected in treated compared to untreated tumours were still observed. Thus, protein clustering using MALDI IMS reflects effective treatment in DLBCL xenografts *in vivo*.

Identification of putative protein candidates from the MALDI data was performed using an approach whereby the significantly deregulated *m*/z ratios reported were matched to *m/z* values from published validated datasets [23], resulting in the list of putatively deregulated proteins reported in Table 1. The results of the proteomic analysis of ibrutinib treated OCI-LY10 tumours demonstrated a significant increase in the levels of a number of proteins inlcuding dermicidin, a pro-survival factor previously linked to an oncogenic function in breast cancer [24], as well a reduction in NEDD8, a small molecule akin to ubiquitin and required in the process of neddylation [25]. Deregulated proteins common to both ibrutinib and idelalisib treatments were members of the thymosin protein family. Thymosin beta-4 (- 2 AA’s, cleaved to seraspenide) and thymosin beta-10, previously implicated as a prognostic marker [26] and determinant of drug sensitivity respectively [27], were found to be significantly upregulated in treated OCI-LY10 xenografts (Figure 5C and 5D). A major advantage of MALDI IMS *in situ* proteomics is the ability to visualize protein distribution in the tissue. Investigation of the proteins thymosin beta-4 and thymosin beta-10 in idelalisib treated OCI-LY10 xenografts demonstrated an increase in the amount of these candidates in the tissue compared to controls (Figure 5E). The overall pattern of distribution within the tumors appeared homogeneous between control and treated samples.

**Table 1:** MALDI IMS proteomics of OCI-LY10 xenografts treated with idelalisib or ibrutinib

Idelalisib				
m/z	Fold change	Protein	Uniprot ID	Pubmed ID
4749,71	1,57	Thymosin beta-4 [Cleaved to Seraspenide]	P62328	21549228
4738,51	1,42	Thymosin beta-10	P63313	21549228
4965,71	1,41	Thymosin beta-4 [Cleaved to Seraspenide]	P20065	15604278

Ibrutinib				
m/z	Fold change	Protein	Uniprot ID	Pubmed ID
4748,85	3,59	Thymosin beta-4 [Cleaved to Seraspenide]	P62328	21549228
4737,87	2,27	Thymosin beta-10	P63313	21549228
2792,49	1,71	Serum Albumin	P02768	22761793
6649,58	1,55	Cytochrome c oxidase subunit 2	P00403	22994484
9263,55	1,51	Dermcidin [Cleaved to DCD-1]	P81605	21549228
8604,43	0,77	NEDD8	Q15843	21549228
8566,42	0,68	Ubiquitin-40S ribosomal protein S27a	P62979	22994484

Protein candidates identified by MALDI IMS as significantly deregulated in OCI-LY10 xenografts following idelalisib or ibrutinib treatment compared to untreated controls (p <0.05).

## DISCUSSION

The potential for early therapy response assessment by combining mutational status with emerging molecular imaging techniques is of great clinical value. Due to the common relapse of DLBCL development of new treatment regimens and improved understanding of their complex cellular effects is required. Herein we have demonstrated that molecular imaging can contribute to very early response assessment to small molecule therapies targeting BCR signaling. Furthermore we demonstrate that molecular imaging allows identification of specific proteomic changes that occur in the lymphoma proteome in response to ibrutinib and idelalisib treatment. The BTK inhibitor ibrutinib has been approved for lymphomas such as CLL [28], mantle cell lymphoma [29] and Waldenström’s disease [30, 31] that critically depend on constitutively activated BCR signaling. Results from a phase 2 study of ibrutinib have furthermore revealed promising results in a subgroup of ABC DLBCL, where the mutational status of pathways involved in NF-κB activation may be useful for predicting response to BTK inhibition. However, as outlined by these data, sequencing analysis alone may not be sufficient to adequately predict response to ibrutinib [8]. It is furthermore established that resistance to ibrutinib can occur, for example in the case of upregulation of the catalytic subunit CD79B, leading to over activation of AKT/MAPK signaling [32]. Activation of CARMA1 has been shown to regulate key signaling processes in ABC DLBCL, including NF-κB, β-catenin and AP-1 complex activation [33, 34]. Accordingly, mutations in the CARMA1 component of the BCR pathway can lead to ibrutinib resistance in ABC DLBCL [35] requiring the application of combination therapies to overcome resistance to treatment [36]. Determination of such mechanisms conferring therapeutic resistance highlights the importance in better defining mutational subtypes to predict patient response and consequently tailor therapy. Alternatively idelalisib, a specific PI3Kδ inhibitor, has also been approved for use in CLL [37] in combination with rituximab and also as a monotherapy in the treatment of both relapsed follicular lymphoma and small lymphocytic lymphoma [11]. To date however, there is no published data regarding treatment of DLBCL with idelalisib.

The established and routine clinical imaging modality^18^F-FDG-PET and additionally ^18^F-FLT-PET are able to provide both response monitoring and survival prediction *in vivo* [38, 39] and were used herein to assess the response of DLBCL xenografts to ibrutinib or idelalisib treatment. As anticipated based on mutation status TBRs of OCI-LY10 xenografts demonstrated significant reductions in response to both BTK and PI3K inhibition. We however observed somewhat discordantly with the mutation status of U2932 xenografts a significant reduction in TBR in response to both inhibitors, therefore it must be considered that additional factors beyond BCR signaling may also play a role in therapy response. We furthermore discovered that SU-DHL-6 cells, serving as an alternative GCB DLBCL cell line, exhibited a change in cell viability upon idelalisib treatment, suggesting that in fact GCB DLBCL may have partial reliance upon BCR signaling. Despite the general consensus that BCR signaling is predominantly a requirement for ABC DLBCL as opposed to GCB DLBCL, GCB cell lines have been shown to rely upon spleen tyrosine kinase (SYK) [40] and cell lines including SU-DHL-16 have previously exhibited significant reductions in cell viability upon ibrutinib treatment [41]. Together these results therefore confirm the utility and importance of mutation status to anticipate therapy response and also outline a potential role of BCR signaling in GCB DLBCL. Additionally, the predictive value added by ^18^F-FDG-PET in this study highlights the advantage of combining a non-invasive established imaging modality with mutation status to predict tumor response very early after treatment initiation.

In addition to the predictive value of early ^18^F-FDG-PET imaging we also postulated that a distinct proteomic profile would manifest in tumors following either BTK or PI3K inhibition. Elucidating the protein profiles of DLBCL subtypes is of significant value with the potential for biomarker discovery, complimenting current methods such as the International Prognostic Index (IPI) that assigns risk but is unable to predict the relapse or refractory nature of DLBCL. Previous DLBCL proteomic studies have used either fixed or frozen tissue samples [42, 43] or employed serum and plasma profiling methods [44-47]. In order to conduct a proteomic investigation of BCR inhibition we have used the in situ imaging technique MALDI IMS as an additional imaging method to identify deregulated proteins. Indeed, MALDI IMS has been used to identify biomarker panels, for example in the case of prediction of Paclitaxel response in breast cancer [48] and also in the context of lymphoma to identify biomarkers allowing the distinction of Hodgkin’s cells from those of lymphadenitis [49]. Herein, upon ibrutinib treatment, NEDD8 expression was reduced in OCI-LY10 xenografts. NEDD8 has previously been implicated in DLBCL whereby inhibition of NEDD8-activating enzyme with the inhibitor MLN4924 induced an apoptotic response in preclinical models of ABC and GCB DLBCL [25]. Furthermore, in CLL, treatment utilizing MLN4924 combined with either idelalisib or ibrutinib led to increased cell death, whereas treatment with idelalisib or ibrutinib alone was unable to exert an effect upon cells [50]. Indeed, to overcome the resistance to ibrutinib treatment reported in clinical trials [8] combination therapies have been shown to exert complimentary effects resulting in apoptosis [51, 52]. The known link between the targets identified with DLBCL in the literature confirms that the proteins identified in the MALDI IMS screen are indeed relevant to BCR pathway inhibition and may present novel options for combination therapies and pathway targeting. Proteins that were deregulated in OCI-LY10 xenografts common to both ibrutinib and idelalisib treatment included thymosin beta-4 and thymosin beta-10 that have been established as mediating a variety of cellular processes including angiogenesis [53], inflammation [54], apoptosis [55] and carcinogenesis [56]. The presence of these markers in the context of BCR function is unclear, however they have been shown to have involvement in NF-κB signaling [57] and consequently potentially represent novel molecules of interest in BCR signaling or DLBCL therapy response.

Herein we have demonstrated that changes in the proteomic profile of lymphoma xenografts treated with the BCR pathway inhibitors ibrutinib and idelalisib can be identified using the *in situ* mass spectrometry technique MALDI IMS. We have also demonstrated using PET imaging that a very early treatment response to both BTK and PI3K inhibition can be measured *in vivo*, not only in DLBCL xenograft models, but also in a syngeneic E*μ-Myc*/BCR^HEL^/sHEL model. We propose that in addition to the indication that mutational status of critical pathogenic pathways provides, that molecular imaging techniques including ^18^F-FDG-PET and MALDI IMS can be used to aid prediction and characterization of therapy response *in vivo* very early upon treatment initiation.

## MATERIALS AND METHODS

### Cell lines

The following cell lines were used: OCI-LY10 (ABC DLBCL), U2932 (ABC DLBCL), SU-DHL-6 (GCB DLBCL) and E*μ-Myc*/BCR^HEL^/sHEL cells [22] were obtained from DMSZ or ATCC. U2932 and SU-DHL-6 were cultured in RPMI 1640 medium containing 10% fetal bovine serum and OCI-LY10 in IMDM with 20% human serum. E*μ-Myc*/BCR^HEL^/sHEL cells were grown in RPMI 1640 medium containing 20% fetal bovine serum [22].

### Mice and tumour xenograft experiments

Animal studies were performed in agreement with the Guide for Care and Use of Laboratory Animals published by the US National Institutes of Health (NIH Publication No. 85-23, revised 1996), in compliance with the German law on the protection of animals, and with approval of the responsible regional authorities (Regierung von Oberbayern). 6-8 week old female CB-17 SCID, NOD SCID or C57BL/6 were obtained from Charles River Laboratories. For induction of xenograft tumours 10 × 10^6^ U2932 and OCI-LY10 or 3 × 10^6^ SU-DHL-6 cells suspended in sterile PBS were injected subcutaneously into the left and right shoulder region. The E*μ-Myc*/BCR^HEL^/sHEL cell line [22] was injected into sub lethally irradiated C57BL/6 mice via tail vein injection.

### Tumour volume and therapeutic regimens

Lymphoma bearing animals were treated daily with idelalisib (3mg/kg p.o.), ibrutinib (25mg/kg p.o.) or carrier (PBS p.o.). Treatment was performed when the xenotransplants reached a size of approximately 500mm³.

### Immunohistochemistry

Tumour xenografts were removed, fixed in formalin and embedded in paraffin. For immunohistochemistry 2-μm sections were deparaffinized and antigen retrieval was performed by pressure cooking in citrate buffer (pH 6) for 7 minutes. Antibody detection of cleaved caspase-3 (Cell Signalling, #9664) was performed using the Dako REAL detection kit (Dako, Glostrup, Denmark) according to the manufacturer’s protocol.

### MTT assay

MTT assays were performed according to manufacturer’s instructions (Promega). Briefly, 10^4^ cells per well (96-well plate) were incubated at 37°C with different concentrations of inhibitors for 48hr. 20μl of MTT dilution (3-(4,5-dimethylthiazol-2-yl)-2,5-diphenyltetrazoliumbromide in PBS) was added per well and cells were incubated for 90 minutes. Absorbance was measured at 490nm using a BioTek ELx800™ Series Universal Microplate Reader (BioTek, VT, USA).

### Flow cytometry for cell cycle analysis

Lymphoma cells were incubated with inhibitors at 37°C. After 48 hours cells were harvested and stored in 70% ethanol at -20°C. On the day of analysis cells were resuspended in PBS. After RNA digestion by ribonuclease A (Sigma-Aldrich 95%), propidium iodide (Sigma-Aldrich) was added in a 50ng/ml dilution. Following incubation for 30 minutes at 37°C cells were analyzed by flow cytometry using a Cyan LX™ flow cytometer (DakoCytomation, CO, USA).

### Cell growth analysis

For growth analysis cells were seeded at 0.5 × 10^6^ cells/ml in 6-well plates and ibrutinib or idelalisib were applied at the doses indicated. Each day the cell concentration was re-adjusted to the original seeding density. Fold growth was calculated as the cumulative growth factor during the course of the experiment.

### PET imaging studies in mice

2-deoxy-2-[18F]fluoro-D-glucose (^18^F-FDG) and 3’-deoxy-3’[^18^F]-fluorothymidine (^18^F-FLT) was synthesized by the Radiopharmacy Unit, TU München. Imaging was performed using a micro PET system (Inveon, SIEMENS Preclinical Solutions, TN, USA), ^18^F-FDG or ^18^F-FLT was administered via tail vein injection at an activity dose of 5-10MBq per mouse. The accumulation of radiotracer in the tumour was allowed for 60 minutes. Mice were then imaged for a 15 minute static acquisition.

### PET data analysis

Analysis was performed using the Inveon Research Workplace (Siemens Healthineers, USA) to semi-quantitatively assess the accumulation of tracer in the tumour. To determine tumour-background ratios (TBRs) three dimensional regions of interest (ROIs) were drawn manually around xenograft tumours and a threshold algorithm (50% maximum intensity – minimum intensity) was applied to each ROI to obtain an intensity value from the area with the highest tumour activity. Background activity was determined by establishing two identically sized 3D ROIs in the spinal muscles below the kidneys.

### MALDI IMS

Xenografts were excised, immediately snap frozen and stored at -80°C. Indium tin oxide glass slides (Bruker Daltonik, 237001) were coated with 20μl 1:1 Poly-Lysine (Sigma, P8920) solution in dH_2_0 with 1μl Nonidet P-40 (Sigma, 74385). Tissue sections were cut at a thickness of 12μm in a pre-cooled cryotome taking extreme care not to thaw samples.

For MSI of tumour cryosections, tissue sections were dried and scanned using a flatbed scanner to acquire digital images for coregistration. Subsequently, the slices were coated with matrix composed of 7 g/l CHCA (Sigma-Aldrich, Taufkirchen, Germany) in 70% methanol, 0.2% trifluoroacetic acid (TFA, Applied Biosystems, Darmstadt, Germany) using an ImagePrep spray device (Bruker Daltonik GmbH, Bremen, Germany) according to the instructions of the manufacturer.

MALDI MSI measurements were carried out on an Ultraflex III mass spectrometer (Bruker Daltonik GmbH, Bremen, Germany) at a spatial resolution of 70 μm in positive reflectron mode with a sampling rate of 1.0 GS/s. For each position measured, a total of 200 laser shots were accumulated. For data generation, the software packages FlexImaging 3.0 and FlexControl 3.0 (Bruker Daltonik GmbH, Bremen, Germany) were used.

In order to assign protein identities to the significantly deregulated candidates identified by MALDI proteomics deregulated *m*/z ratios reported were matched to *m/z* values from published validated datasets [23]. Heatmaps were generated using Metaboanalyst 3.0 using the following settings: mass tolerance 0.025, relative time tolerance 30, log transformation, distance measure: Euclidian, clustering algorithm: Ward.

### Statistical analysis

All statistical tests were performed using GraphPad Prism (GraphPad Software, CA, USA). P-values < 0.05 were considered statistically significant. Quantitative values were expressed as mean ± standard deviation (SD).

## Abbreviations

AA: Amino acid
ABC: Activated B-cell type
BCR: B-cell receptor
BTK: Bruton’s tyrosine kinase
CLL: Chronic lymphocytic leukaemia
DLBCL: Diffuse large B-cell lymphoma
GCB: Germinal center B-cell
H&E: Hematoxylin and Eosin
IMS: Imaging mass spectrometry
FDG: 2-deoxy-2-[18F]fluoro-D-glucose
FLT: 3’-deoxy-3’[^18^F]-fluorothymidine
MALDI: Matrix assisted laser desorption ionization
MTT: 3-(4,5-dimethylthiazol-2-yl)-2,5-dipheny ltetrazoliumbromide
m/z: mass-to-charge ratio
NHL: Non-Hodgkin lymphoma
PET: Positron emission tomography
PBS: Phosphate buffered saline
p.o.: Per oral
PTM: Post-translational modification
ROI: Region of interest
SD: Standard deviation
TBR: Tumour-to-background ratio
